# Activation of Recombinantly Expressed l-Amino Acid Oxidase from *Rhizoctonia solani* by Sodium Dodecyl Sulfate

**DOI:** 10.3390/molecules22122272

**Published:** 2017-12-20

**Authors:** Katharina Hahn, Yvonne Hertle, Svenja Bloess, Tilman Kottke, Thomas Hellweg, Gabriele Fischer von Mollard

**Affiliations:** 1 Biochemistry III, Department of Chemistry, Universitätsstrasse 25, Bielefeld University, 33615 Bielefeld, Germany; katharina.hahn@uni-bielefeld.de (K.H.); sbloess@uni-bielefeld.de (S.B.); 2 Physical and Biophysical Chemistry, Department of Chemistry, Universitätsstrasse 25, Bielefeld University, 33615 Bielefeld, Germany; yvonne.hertle@uni-bielefeld.de (Y.H.); tilman.kottke@uni-bielefeld.de (T.K.); thomas.hellweg@uni-bielefeld.de (T.H.)

**Keywords:** l-amino acid oxidase, activation, SDS, active conformation, conformational change, photon correlation spectroscopy (PCS)

## Abstract

l-Amino acid oxidases (l-AAO) catalyze the oxidative deamination of l-amino acids to the corresponding α-keto acids. The non-covalently bound cofactor FAD is reoxidized by oxygen under formation of hydrogen peroxide. We expressed an active l-AAO from the fungus *Rhizoctonia solani* as a fusion protein in *E. coli*. Treatment with small amounts of the detergent sodium dodecyl sulfate (SDS) stimulated the activity of the enzyme strongly. Here, we investigated whether other detergents and amphiphilic molecules activate 9His-*rs*LAAO1. We found that 9His-*rs*LAAO1 was also activated by sodium tetradecyl sulfate. Other detergents and fatty acids were not effective. Moreover, effects of SDS on the oligomerization state and the protein structure were analyzed. Native and SDS-activated 9His-*rs*LAAO1 behaved as dimers by size-exclusion chromatography. SDS treatment induced an increase in hydrodynamic radius as observed by size-exclusion chromatography and dynamic light scattering. The activated enzyme showed accelerated thermal inactivation and an exposure of additional protease sites. Changes in tryptophan fluorescence point to a more hydrophilic environment. Moreover, FAD fluorescence increased and a lower concentration of sulfites was sufficient to form adducts with FAD. Taken together, these data point towards a more open conformation of SDS-activated l-amino acid oxidase facilitating access to the active site.

## 1. Introduction

l-Amino acid oxidases (l-AAO, EC 1.4.3.2) have been identified in organisms from different kingdoms such as bacteria, algae, fungi and snakes [1,2,3,4]. They catalyze the oxidative deamination of l-amino acids forming the corresponding α-keto acids, ammonia and hydrogen peroxide. This reaction requires the cofactor flavin adenine dinucleotide (FAD), which is tightly bound but not covalently attached to the enzyme. The oxidized form of FAD accepts a hydride, converting l-amino acids to α-imino acids, which hydrolyse spontaneously to α-keto acids and ammonia. FAD is regenerated by oxygen under emergence of hydrogen peroxide in the oxidative half-reaction. Most l-AAOs form dimers, but active monomers and dimers have been described for the same l-AAO from the fungus *Trichoderma harzianum* ETS323, depending on growth conditions [4,5].

The related d-amino acid oxidases are already used for biotechnological applications [6]. l-Amino acid oxidases could be used for the production of enantiomerically pure d-amino acids from racemic mixtures of α-amino acids by enzymatic resolution. d-amino acids are building blocks for many antibiotics and pharmaceuticals and therefore have gained significance in the pharmaceutical industry [7]. Many α-keto acids are precursors of their corresponding proteinogenic l-amino acids and could therefore be produced by fermentation in genetically engineered microorganisms [8]. However, the corresponding α-keto acids are not intermediates in the biosynthesis of l-cysteine, l-methionine, l-arginine and l-lysine. These α-keto acids as well as α-keto acids without corresponding proteinogenic l-amino acids could be synthesized with the help of l-amino acid oxidases. In addition, l-AAOs might be used as components of biosensors for l-amino acids.

Biotechnological applications of l-amino acid oxidases with broad substrate spectrum have not been possible so far because the recombinant expression of sufficient amounts of these enzymes has not been achieved [4,9]. Expression of the l-AAO from the bacterium *Rhodococcus opacus* in *E. coli* resulted in inclusion bodies, which did not yield active enzyme after refolding attempts [10]. The l-AAO from *Streptococcus oligofermentans* has been expressed as soluble 6His-tagged fusion protein in *E. coli* [11]. The expression of an l-AAO from the fungus *Hebeloma cylindrosporum* in *E. coli* as a soluble 6His-tagged fusion protein was successful with low yields because it was almost completely in inclusion bodies [12]. However, this enzyme had to be activated with low amounts of the detergent sodium dodecyl sulfate (SDS). 

Recently, we have succeeded in expressing an active l-AAO from the fungus *Rhizoctonia solani* as a fusion protein with maltose-binding protein (MBP) as a solubility tag in *E. coli* [13]. This enzyme converted several large hydrophobic and basic l-amino acids. Methyl esters of these l-amino acids also served as substrates. This result is interesting for technical applications because esters of α-keto acids are more stable than the corresponding free α-keto acids. After incorporation of nine histidine residues as a second affinity tag (MBP-P-9His-*rs*LAAO1), a 1 L *E. coli* culture yielded 4.4 mg pure enzyme after a two-step affinity chromatographic purification including the proteolytic removal of the MBP tag. The enzymatic activity towards all substrates was stimulated by a factor of 50–100 by low amounts of SDS, which is known to denature proteins. This treatment resulted in a specific activity of 9 U∙mg^−1^ after activation with 1 mM SDS. While SDS increased the *v*_max_ strongly, the *K*_m_ value was barely influenced for the seven substrates tested, suggesting that the binding of the substrate in the active site was not influenced. 

Here, we investigate whether other substances can cause this activation, whether the enzyme is a dimer and whether structural changes can be detected and characterized upon activation.

## 2. Results

### 2.1. Effects of Detergents and Fatty Acids on 9His-rsLAAO1 Activity

**Figure 1:**
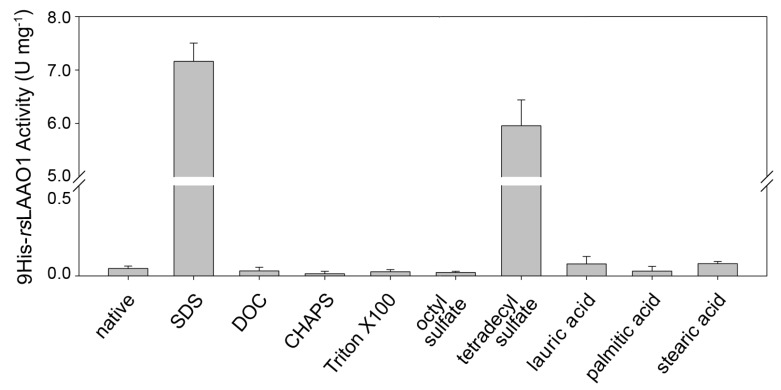
9His-*rs*LAAO1 expressed in *E. coli* was activated by long-chain alkyl sulfates. 9His-*rs*LAAO1 (0.1 mg∙mL^−1^) was preincubated for 10 min with 1 mM of the detergents sodium dodecyl sulfate (SDS), deoxycholate (DOC), CHAPS, Triton X-100, sodium octyl sulfate (C_8_) or sodium tetradecyl sulfate (C_14_), or 1.5 mM of the fatty acids lauric acid, palmitic acid or stearic acid. These samples were diluted into the peroxidase/*o*-dianisidine assay solution with 10 mM arginine as substrate and H_2_O_2_ production was measured. Data are mean of at least three independent experiments (native *n* = 9; SDS *n* = 11, sodium octyl sulfate and sodium tetradecyl sulfate *n* = 8), error bars are standard deviations (SD).

Recombinantly expressed and purified MBP-*rs*LAAO1 and 9His-*rs*LAAO1 can be strongly activated by 1 mM SDS as described earlier [13]. First, we examined whether other detergents or fatty acids are able to activate purified 9His-*rs*LAAO1. MBP-P-9His-*rs*LAAO1 was expressed in the *E. coli* strain Arctic Express at 11 °C, cells were lysed and the soluble fractions applied to amylose resin. After digestion with PreScission protease, 9His-*rs*LAAO1 was further purified using Ni^2+^-NTA resin. 0.1 mg∙mL^−1^ 9His-*rs*LAAO1 was preincubated with 1 mM SDS, the anionic detergent deoxycholate, the zwitterionic detergent CHAPS (3-[(3-cholamidopropyl)dimethylammonio]-1-propanesulfonate) or the nonionic detergent Triton X-100. Afterwards, enzymatic activity was assayed using arginine as substrate and *o*-dianisidine and peroxidase to measure initial production of hydrogen peroxide. Deoxycholate (DOC), CHAPS or Triton X-100 did not stimulate the activity of 9His-*rs*LAAO1 compared to untreated samples (Figure 1).

As SDS was strongly activating, we checked for the effect of an alkyl sulfate with a shorter and a longer alkyl chain than SDS (C_12_). Sodium octyl sulfate (C_8_) did not activate 9His-*rs*LAAO1 (Figure 1). 1 mM sodium tetradecyl sulfate (C_14_) strongly activated 9His-*rs*LAAO1, however, it was less effective than SDS at the same concentration. To investigate whether a carboxylic group can replace the sulfate group, the fatty acids dodecanoic acid (lauric acid, C_12_), hexadecanoic acid (palmitic acid, C_16_) and octadecanoic acid (stearic acid, C_18_) were also tested for their effect on 9His-*rs*LAAO1. These fatty acids had no or minor stimulatory effects on the enzymatic activity (Figure 1). 

### 2.2. 9His-rsLAAO1 Behaved as a Dimer in the Native and SDS-Activated State

**Figure 2:**
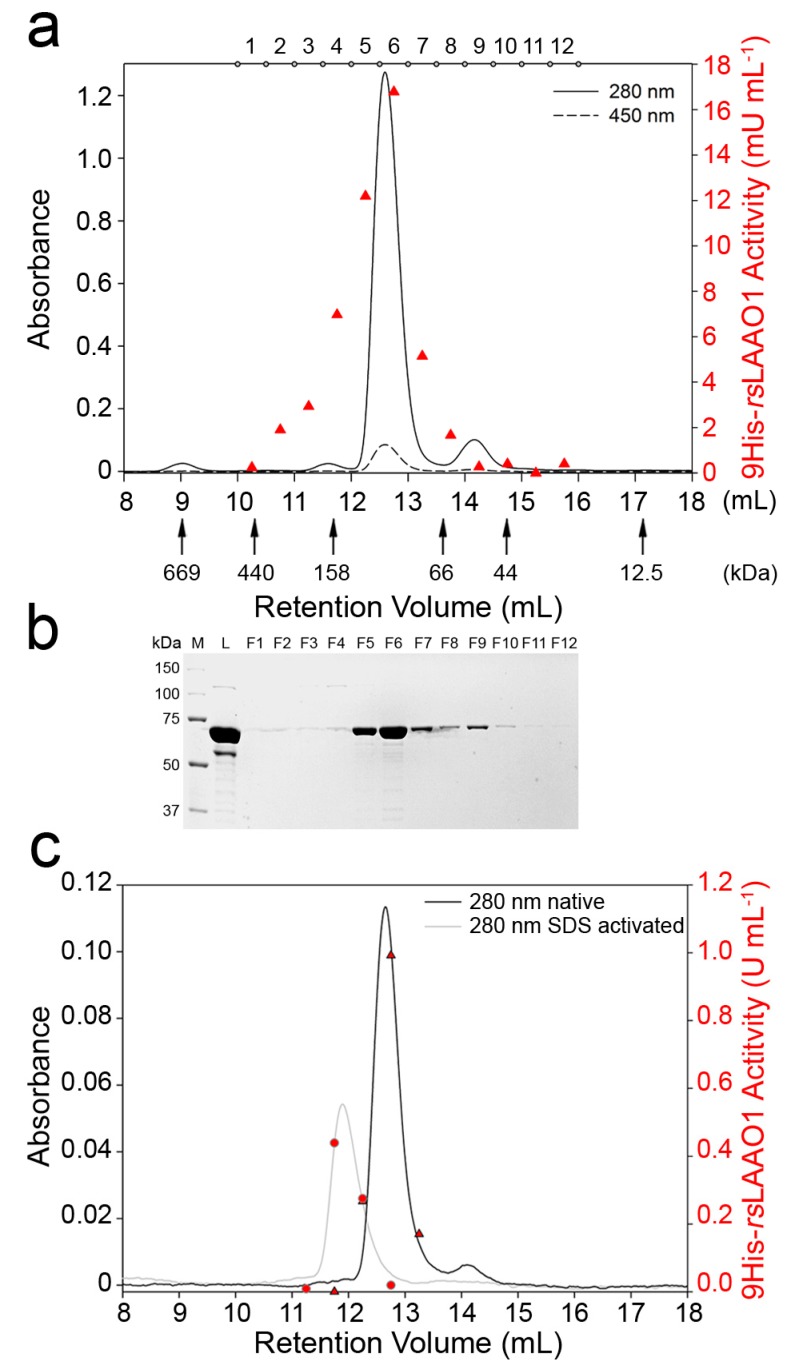
9His-*rs*LAAO1 behaved as a dimer in size-exclusion chromatography, which increased in hydrodynamic radius after SDS activation. (**a**) Purified 9His-*rs*LAAO1 was separated on a Superdex 200 *Increase* 10/300 GL column. Most 9His-*rs*LAAO1 was detected at 12.6 mL elution volume equivalent to a molecular mass of 120 kDa consistent with a dimer of 9His-*rs*LAAO1. A minor peak was detected at an elution volume equivalent to 55 kDa and assigned to a monomer. Solid line: protein absorbance at 280 nm; dotted line: FAD absorbance at 450 nm; red triangles: LAAO activity of fractions 1–12 measured using a coupled peroxidase *o*-dianisidine assay without SDS activation; (**b**) Column load (L) and fractions 1–12 were separated by SDS–PAGE (polyacrylamide gel electrophoresis) and stained with Coomassie; (**c**) 9His-*rs*LAAO1 activated by 1.5 mM SDS (grey line) eluted earlier than native 9His-*rs*LAAO1 (black line). Enzymatic activity was measured directly for the SDS-activated 9His-*rs*LAAO1 (red circles) or after SDS activation for native 9His-*rs*LAAO1 (red triangles).

Most l-amino acid oxidases are active only as homodimers [4]. However, an l-AAO from the fungus *Trichoderma harzianum* ETS323 is secreted as an active monomeric or dimeric form depending on cultivation conditions [5]. Therefore, we analyzed the dimerization state of recombinant purified 9His-*rs*LAAO1 by size-exclusion chromatography (SEC). The major protein peak of native protein was detected at an elution volume of 12.6 mL (Figure 2a). 

A minor FAD-containing peak was found at an elution volume of 14.2 mL corresponding to an apparent molecular mass of 55 kDa according to comparisons with protein standards. Both peaks contain intact 9His-*rs*LAAO1 as seen by SDS–PAGE (Figure 2b). These data indicate that the minor peak at 14.2 mL corresponds to the monomer. The deviation from the calculated molecular mass of 75 kDa could be due to the shape of the protein or due to interaction with the matrix. The major peak eluted at a position corresponding to an apparent molecular mass of 120 kDa. As this apparent molecular mass is about twice that of the monomer, the predominant form was a dimer. The dimeric 9His-*rs*LAAO1 was subjected to a second size-exclusion chromatography, yielding a single peak at the same elution volume, indicating that the dimer is not dynamic but stable (data not shown).

Enzymatic activity was assayed without SDS activation. The highest activity was detected in the major protein peak (Figure 2a). Interestingly, substantial activity was also detected in fractions 3 and 4, even though the protein concentration was very low. Therefore, the specific activity (activity per mg protein) in these fractions was higher than in the dimer peak. Specific activities were comparable if the fractions were assayed after SDS activation (Figure 2c, triangles). These data indicate that a more active conformation with a slightly larger size might exist. Next, SDS-activated 9His-*rs*LAAO1 was separated by size-exclusion chromatography. A major peak was found at an elution volume of 11.9 mL corresponding to 168 kDa (Figure 2c). These data indicate that 9His-*rs*LAAO1 does not oligomerize but that the dimer increases slightly in size upon SDS activation.

Photon correlation spectroscopy (PCS) was performed to analyze the diffusion and hydrodynamic radius of the native and SDS-activated 9His-*rs*LAAO1 in more detail. Therefore, the scattering intensity of the sample was measured at different angles and analyzed by the formalism of auto-correlation functions [14] and CONTIN [15,16] analysis. The obtained mean relaxation rate $\bar{\Gamma }$ is connected to the diffusion coefficient of the protein in solution and can further be converted into the hydrodynamic radius $R_{h}$. For more details, see experimental section. 

**Figure 3:**
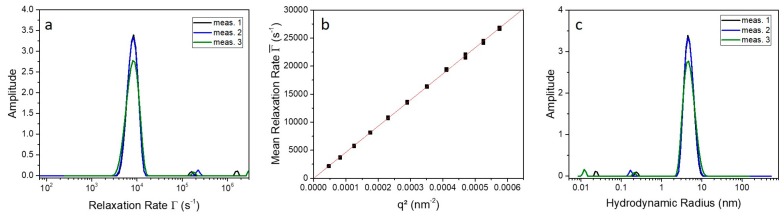
(**a**) Relaxation rate distribution of three individual measurements of the native 9His-*rs*LAAO1 protein measured at an angle of 60° and a temperature of 20 °C; (**b**) Mean relaxation rate as a function of $q^{2}$; (**c**) Determined size distribution of native 9His-*rs*LAAO1 at 20 °C and a scattering angle of 60°.

For an example, the mean relaxation rate $\bar{\Gamma }$ and the radius distribution at an angle of 60° as well as the $\bar{\Gamma }$ vs. $q^{2}$ plot of the native 9His-*rs*LAAO1 are shown in Figure 3. In the relaxation rate distribution (Figure 3a), one main peak is observable; the smaller peaks at high Γ-values are artefacts caused by the numerical data analysis. The presence of only one monomodal narrow peak corresponds to only one diffusing species in solution with a low polydispersity. If there are particles with only translational diffusion, a plot of $\bar{\Gamma }$ vs. $q^{2}$ should result in a linear dependence through the origin. Since this assumption is fulfilled (Figure 3b), the mean relaxation rate can be converted to the hydrodynamic radius via the diffusion coefficient $D^{T}$ and using Equation (5). The calculations resulted in a hydrodynamic radius for the native 9His-*rs*LAAO1 of about 4.6 nm. This radius is the radius of a sphere, which behaves hydrodynamically equivalent to the 9His-*rs*LAAO1 in solution.

It has to be mentioned here that the 9His-*rs*LAAO1 monomer and dimer do not differ too much in hydrodynamic behavior and consequently do not appear as two separated peaks in the relaxation rate distribution, in contrast to the results from size-exclusion chromatography (SEC).

Based on a globular structure and the molecular weight of the protein, a theoretical hydrodynamic radius can also be calculated for comparison using the following equation.

$$R^{theo}=\frac{1}{2}{\left(\frac{6M\nu }{\pi N_{A}}\right)}^{1/3}$$

Here, M is the molecular weight of the protein and $N_{A}$ the Avogadro number. Further, we used for the partial specific volume of the protein ν= 0.73 mL∙g^−1^ [17]. Therefore, the theoretical hydrodynamic radius for 9His-*rs*LAAO1 can be estimated using the calculated and SEC-determined molecular weight and the results are summarized in Table 1. The thickness of the hydration layer was assumed to be 0.3 nm as found in most hydrodynamic studies [18,19].

The ratio of the experimental $R_{h}$ value and the computed $R_{h}^{theo}$ value allows determination of the anisotropy of the 9His-*rs*LAAO1 molecule. In the present case, for the native 9His-*rs*LAAO1, we obtain $R_{h}/R_{h,SEC}^{theo}=1.3$. For a globular protein this value would be one. This underlines the assumption that the protein exhibits an elongated structure comparable to a dumbbell. 

**Figure 4:**
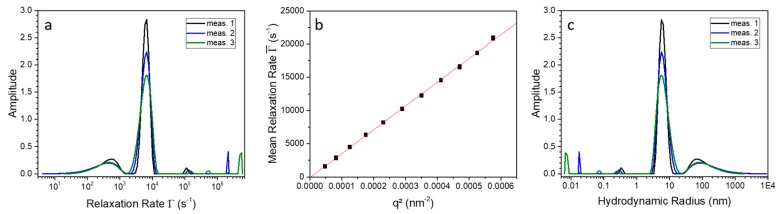
(**a**) Relaxation rate distribution of three individual measurements of the SDS-activated 9His-*rs*LAAO1 protein measured at an angle of 60° and a temperature of 20 °C; (**b**) Mean relaxation rate as a function of $q^{2}$; (**c**) Determined size distribution of SDS-activated 9His-*rs*LAAO1 at 20 °C and a scattering angle of 60°.

To verify the influence of SDS on 9His-*rs*LAAO1, the PCS measurements have been repeated with the protein in the presence of 1.5 mM SDS (critical micelle concentration of SDS = 8.2 mM at 298 K) [20]. In contrast to Figure 3a for the native protein, the measurements in the presence of SDS (Figure 4) show an additional broad peak beside the mean relaxation rate, the major peak at 6000 s^−1^. That indicates a dynamic formation of aggregates, since the sample was filtered and centrifuged before measurement to eliminate stable aggregates. Here, it has to be mentioned that in terms of particle number these aggregates do not represent any significant amount of particles, because the scattering intensity is proportional to the power of six of the radius. An evaluation of the mean relaxation rate shows that pure translation diffusion of the protein is observed. The corresponding hydrodynamic radius of the 9His-*rs*LAAO1 dimer after SDS activation is about 6.0 nm. An analogue determination of the second broad peak resulted also in a linear dependence. Hence, this second mode corresponds to diffusion of spurious amounts of aggregates and not to a rotational mode of the 9His-*rs*LAAO1 dimer (data not shown). 

The increase of the hydrodynamic radius from 4.6 nm to 6.0 nm upon addition of SDS to the protein can be caused by two reasons. First, the SDS molecules can induce conformational changes in the protein structure and therefore the hydrodynamic size can increase. Second, SDS molecules can bind to positive or hydrophobic patches on the 9His-*rs*LAAO1 surface and increase the particle radius. From the literature, it is known that SDS molecules in solution exhibit a length of about 1.36 nm [21].

In summary, two independent methods indicate that SDS activation causes a size increase in 9His-*rs*LAAO1. This could be due to bound SDS or conformational changes.

### 2.3. Indications for Conformational Changes in SDS-Activated 9His-rsLAAO1

Next we investigated whether SDS activation influenced properties of 9His-*rs*LAAO1, which depend on its conformation. Thermal inactivation of the enzymatic activity was assayed as the first parameter. 

**Figure 5:**
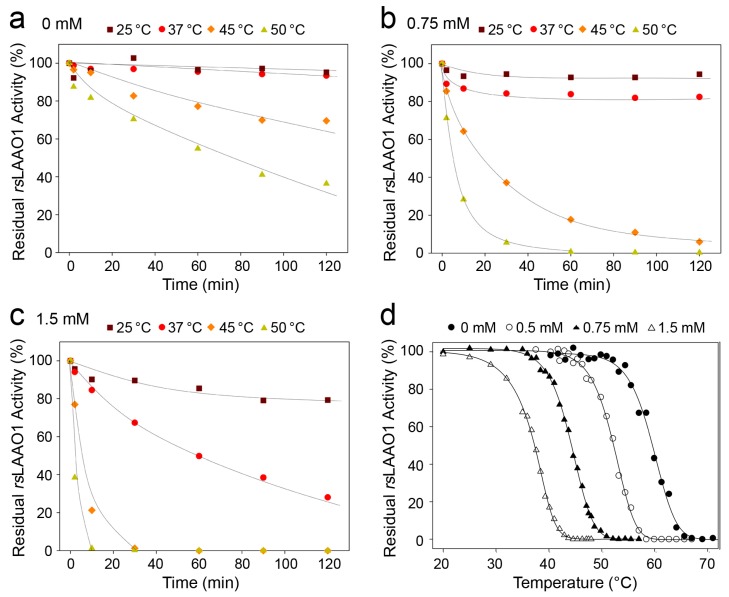
Thermal inactivation of 9His-*rs*LAAO1 was accelerated by increasing concentrations of SDS. Purified 9His-*rs*LAAO1 (0.1 mg∙mL^−1^) was incubated with (**a**) 0 mM SDS; (**b**) 0.75 mM SDS or (**c**) 1.5 mM SDS at 25 °C (purple squares), 37 °C (red circles), 45 °C (orange diamonds) or 50 °C (yellow triangles) for the indicated amount of time and assayed for residual enzymatic activity using a coupled peroxidase *o*-dianisidine assay with arginine as substrate; (**d**) The residual activity of 9His-*rs*LAAO1 (0.1 mg∙mL^−1^) was measured after a preincubation of 10 min at the indicated temperature in the presence of 0 mM (filled circles), 0.5 mM (open circles), 0.75 mM (filled triangles) or 1.5 mM SDS (open triangles).

Purified 9His-*rs*LAAO1 was preincubated for different amounts of time at 25 °C, 37 °C, 45 °C or 50 °C and afterwards assayed for the remaining enzymatic activity. 9His-*rs*LAAO1 was stable for 120 min at 25 °C and 37 °C (Figure 5a). About 70% of the activity remained after 60 min at 45 °C and 50% at 50 °C.

9His-*rs*LAAO1 was inactivated faster in the presence of 0.75 mM SDS. Enzymatic activity was completely lost after 60 min at 50 °C and less than 20% remained after 60 min at 45 °C (Figure 5b). Inactivation was even faster at 45 °C and 50 °C in the presence of 1.5 mM SDS (Figure 5c). 9His-*rs*LAAO1 lost 50% of the activity in 60 min at 37 °C. In a different set of experiments, 9His-*rs*LAAO1 was preincubated for 10 min at different temperatures and afterwards assayed for the residual activity. 50% inactivation of 9His-*rs*LAAO1 was reached at 60 °C in the absence of SDS (Figure 5d). The same degree of inactivation was obtained at 54 °C in the presence of 0.5 mM SDS and at 37.5 °C with 1.5 mM SDS. These data indicate that the thermal inactivation of 9His-*rs*LAAO1 accelerated strongly with increasing SDS concentrations.

**Figure 6:**
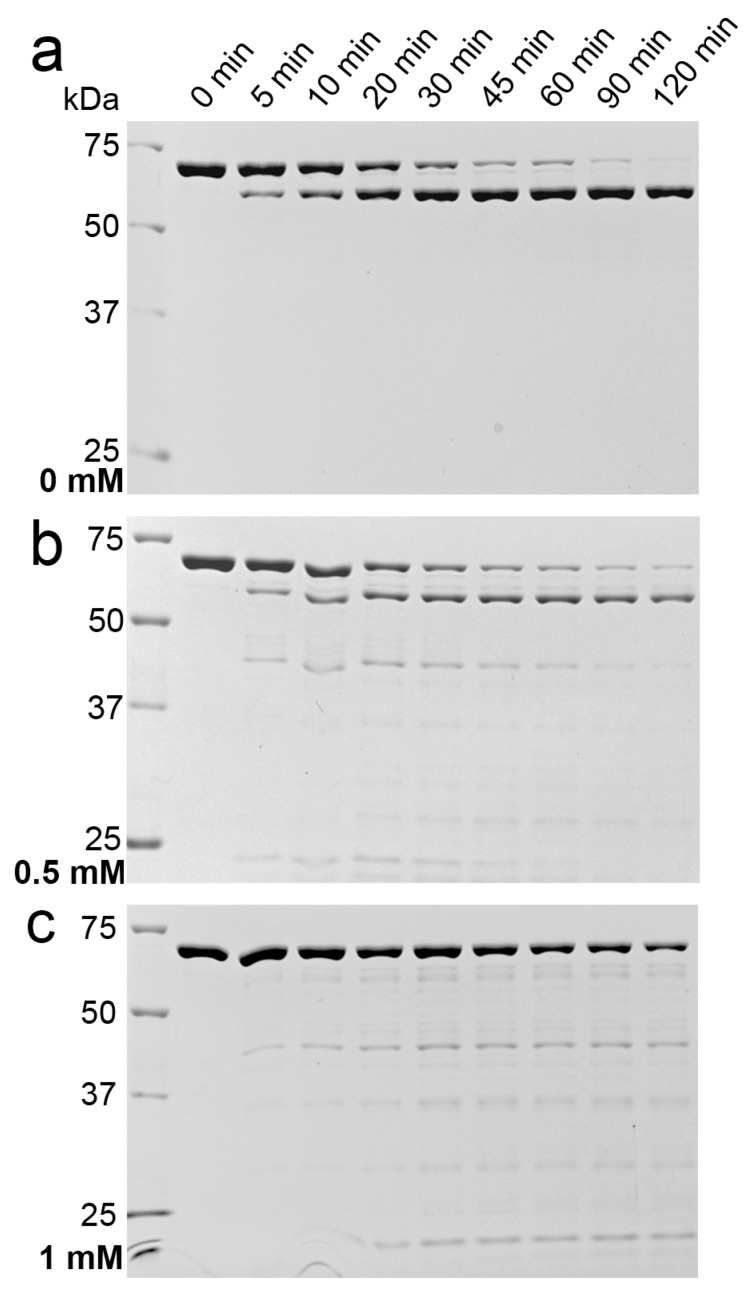
Increased proteolytic sensitivity of 9His-*rs*LAAO1 after SDS treatment. Purified 9His-*rs*LAAO1 (0.3 mg∙mL^−1^) was incubated with 0.3 µg∙mL^−1^ trypsin at 37 °C for the indicated periods of time (**a**) in the absence; (**b**) in the presence of 0.5 mM SDS or (**c**) in the presence of 1 mM SDS. Samples were separated by SDS–PAGE and stained with Coomassie.

The conformation of a protein also determines whether a specific recognition site is accessible for a protease. Native 9His-*rs*LAAO1 was incubated with low amounts of trypsin for different amounts of time up to 120 min and samples were analyzed by SDS–PAGE (Figure 6a).

Intact 9His-*rs*LAAO1 was slowly cleaved to a fragment of 65 kDa, which was stable for 120 min. Trypsin was fully active in 0.5 mM and partially active in 1 mM SDS as seen for the substrates hemoglobin, ovalbumin and bovine serum albumin (Supplementary Figure S1). A different pattern emerged for 9His-*rs*LAAO1 after incubation with trypsin in the presence of 1 mM SDS. Many different low-molecular-mass bands emerged parallel to a reduction in the amount of full-length 9His-*rs*LAAO1 (Figure 6c). However, a stable 65 kDa band was not detected. An intermediate behavior was observed in the presence of 0.5 mM SDS (Figure 6b). Some of the full-length protein was degraded into several bands below 45 kDa at intermediate time points, which were fully degraded after 120 min. A fraction of the enzyme was cleaved to the stable 65 kDa fragment. These data suggest that more trypsin sites became accessible in the presence of SDS. This finding is consistent with a more open conformation.

**Figure 7:**
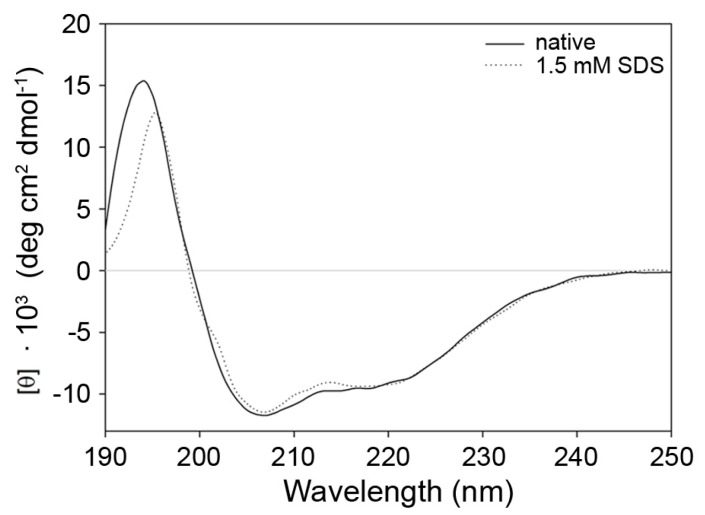
Similar secondary structure of native and SDS-activated 9His-*rs*LAAO1 according to circular dichroism spectroscopy. 9His-*rs*LAAO1 (4 µM) without (solid line) or with 1.5 mM SDS (dotted line) were analyzed by CD spectroscopy in phosphate buffer with 100 mM sodium sulfate at room temperature. Absorption by components of the buffer might cause the deviations between 190 and 195 nm.

Next, different types of spectroscopy were applied to analyze secondary and tertiary structure changes in the protein. Circular dichroism (CD) spectra in the range of 190–260 nm are very specific for different peptide secondary structures. For example, α-helical peptides give rise to characteristic minima in ellipticity at 208 nm and 222 nm, β-sheets at 218 nm. Therefore, CD spectroscopy was used to calculate the secondary structure content. The secondary structure of 9His-*rs*LAAO1 consists of 43% α-helices (standard deviation 2.9%) and 17% β-sheets (standard deviation 3.4%) in five independently purified enzyme preparations according to the CDSSTR algorithm used by the DichroWeb server [22]. The secondary structure content of 9His-*rs*LAAO1 in the presence of 1.5 mM SDS was calculated as 44% α-helices (standard deviation 6.3%, *n* = 5) and 17% β-sheets (standard deviation 3.8%) (Figure 7). 

These data indicate that SDS does not cause major changes in the secondary structure of 9His-*rs*LAAO1. 

**Figure 8:**
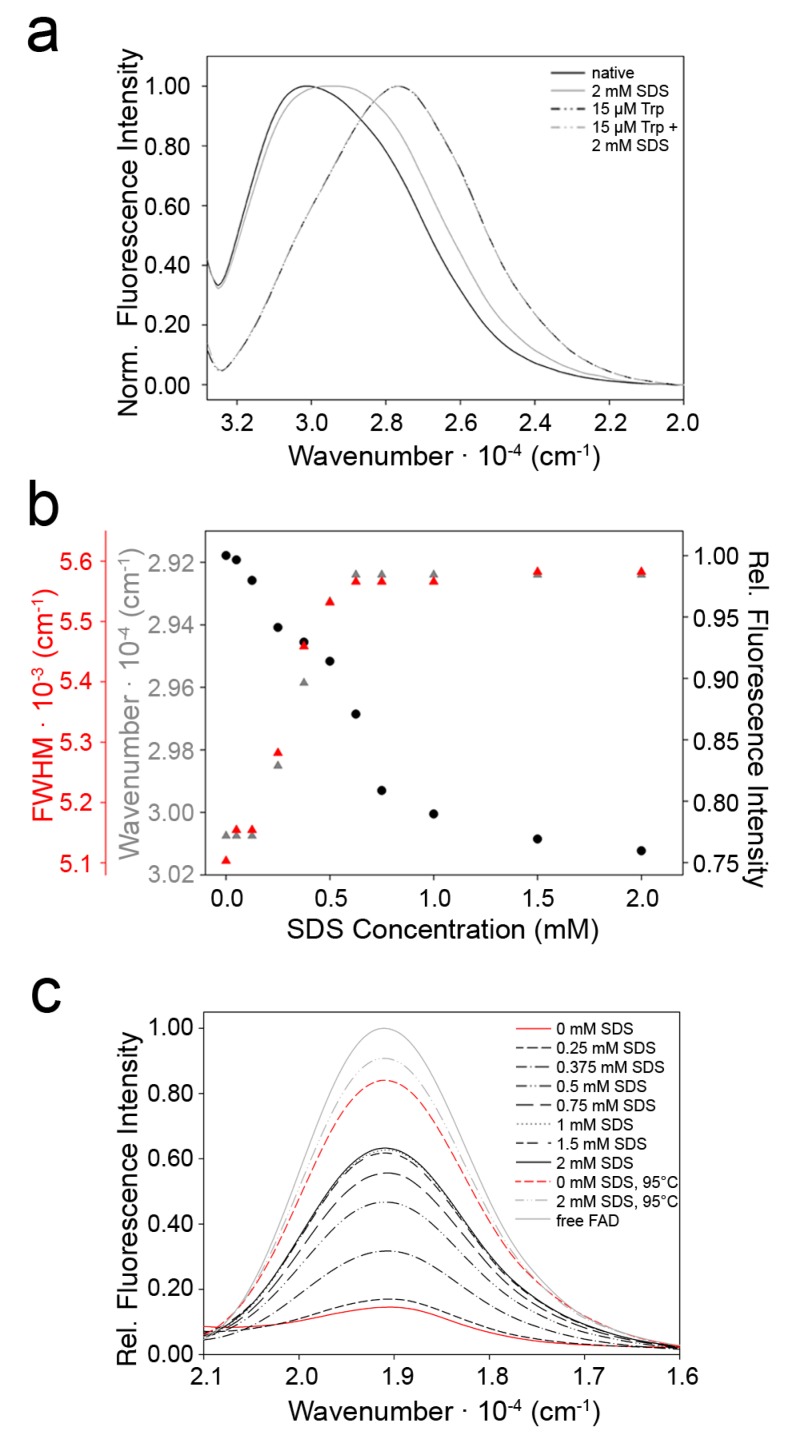
Tryptophan and FAD fluorescence point towards conformational changes in 9His-*rs*LAAO1 upon SDS activation. (**a**) Emission spectra of 9His-*rs*LAAO1 (2 µM) without (solid black line) or with 2 mM SDS (solid grey line) and 15 µM tryptophan without (black dashes) or with 2 mM SDS (grey dashes) after excitation of intrinsic tryptophan fluorescence at 295 nm. Maximal intensity in each spectrum was normalized to 1 to facilitate comparisons of the shape of the peaks; (**b**) Tryptophan fluorescence intensity (black circles), wavenumber of the emission maximum (grey triangles) and the full width at half maximum (FWHM) of the emission peak (red triangles) were quantified from spectra of 9His-*rs*LAAO1 incubated with different concentration of SDS; (**c**) Emission spectra after excitation of FAD fluorescence at 450 nm for 9His-*rs*LAAO1 (3 µM) without SDS (red line) or increasing concentrations (black dashes) up to 2 mM SDS (black line). Spectra of free FAD (grey line) and heat-denatured 9His-*rs*LAAO1 without SDS (red dashes) or with 2 mM SDS (grey dashes) were recorded for comparison.

The fluorescence properties of tryptophan residues depend on the polarity of the surroundings and the position of neighboring amino acid side chains, which influence the wavenumber of the emission maximum and the fluorescence intensity [23]. 9His-*rs*LAAO1 contains 16 tryptophan residues. The emission maximum of native, untreated 9His-*rs*LAAO1 was observed at a higher wavenumber (thus a lower wavelength) than that of tryptophan (Figure 8a). This observation is typical for a protein with tryptophan residues in hydrophobic surroundings. In the presence of 2 mM SDS, the emission maximum shifted partially to lower wavenumbers (higher wavelengths), while this concentration of SDS did not have an effect on the wavenumber of the emission maximum of free tryptophan (Figure 8a). A maximal shift in the emission maximum was reached at 0.7 mM SDS for 9His-*rs*LAAO1 (Figure 8b). In addition, SDS caused a broadening of the fluorescence band by the shift in emission maximum of some of the 16 tryptophans (Figure 8a), which was quantified as an increase in the full width at half maximum (FWHM, Figure 8b). A hydrophobic environment increases the intensity of the tryptophan fluorescence. Accordingly, in 9His-*rs*LAAO1, this fluorescence intensity was reduced by SDS treatment (Figure 8b). Again, little changes occurred above 1 mM SDS corresponding to the maximal activation of 9His-*rs*LAAO1 at 1–2 mM SDS [13].

Taken together, these data indicate that one or more tryptophan residues were exposed to more hydrophilic surroundings by SDS treatment, indicating that a change in tertiary structure had occurred.

The fluorescence spectrum of FAD also depends on the surrounding protein [24]. Free FAD and FAD from heat-denatured 9His-*rs*LAAO1 had a much higher fluorescence intensity than FAD in native 9His-*rs*LAAO1 (Figure 8c). Increasing the concentration of SDS increased the fluorescence intensity of FAD up to a plateau reached at 1 mM SDS. The FAD fluorescence was further increased considerably by heat denaturation in the presence of 2 mM SDS. By contrast, SDS increased the fluorescence intensity only slightly in the heat-denatured sample, indicating that SDS had a minor direct effect on the FAD fluorescence. These data suggest that SDS influences the conformation of the enzyme at the active site, leading to an increase in distance of FAD to the quenching aromatic amino acids.

Amino acid oxidases belong to the class 2 flavoproteins, which produce hydrogen peroxide, may form an FAD anion radical and undergo adduct formation between FAD and sulfites [25,26]. 

**Figure 9:**
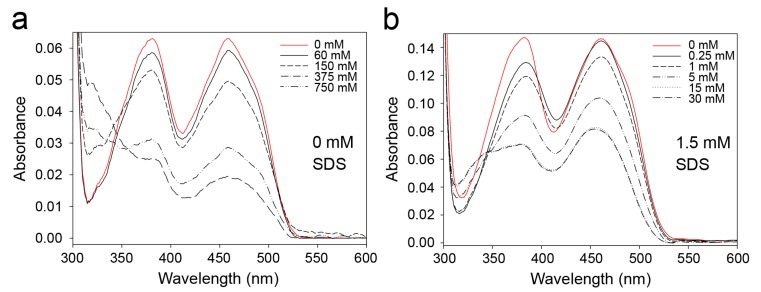
FAD in SDS-activated 9His-*rs*LAAO1 reacted with sulfite at much lower concentrations than FAD in the native enzyme. Absorption spectra were recorded of (**a**) native 9His-*rs*LAAO1 (7.5 µM) or (**b**) 9His-*rs*LAAO1 activated with 1.5 mM SDS (15 µM). The enzymes were incubated with 0–750 mM (native enzyme) or 0–30 mM (SDS-activated 9His-*rs*LAAO1) of sodium sulfite for 15 min at room temperature in the dark. Adduct formation with sulfites reduced the absorbance of FAD at 380 and 450 nm.

Therefore, we tested whether the reactivity towards sulfites in 9His-*rs*LAAO1 is influenced by SDS. Adduct formation with sulfites reduces the absorbance of FAD at 380 and 450 nm. At least 150 mM sulfite was required for a strong reduction in FAD absorption in native 9His-*rs*LAAO1 (Figure 9a). By contrast, adducts were formed already at 5 mM sulfite in 9His-*rs*LAAO1 treated with 1.5 mM SDS (Figure 9b). Again, these data suggest that SDS influenced the conformation at the active site, thereby increasing the accessibility for ionic reagents.

## 3. Discussion

We described earlier that 1 mM SDS activates *rs*LAAO1 [13]. Here, we wanted to determine the origin of this activation and its specificity. After using alkyl sulfates with different chain lengths, we found that sodium tetradecyl sulfate with a C_14_ alkyl chain was also effective but not sodium octyl sulfate with its C_8_ alkyl chain. Deoxycholate, CHAPS and Triton X-100 did not activate 9His-*rs*LAAO1. These three detergents do not influence a poly phenol oxidase from saffron [27] and phenol oxidases from cockroach and horseshoe crab [28,29], which are also activated by SDS. By contrast, tyrosinase from Xenopus can be activated by deoxycholate and SDS, but not by Triton X-100 [30]. The phenol oxidases from cockroach and horseshoe crab mentioned above [28,29] and a poly phenol oxidase from spinach [31] can be activated by fatty acids. By contrast, 9His-*rs*LAAO1 expressed in *E. coli* was barely influenced by fatty acids. These data show that both a sulfate group and a long alkyl chain are required for efficient activation of 9His-*rs*LAAO1, while some phenol oxidases, poly phenol oxidases and tyrosinases can be activated by a broader range of amphiphilic molecules.

A possible mechanism of activation is a change in the dimerization or oligomerization state of an enzyme. However, the main peak of 9His-*rs*LAAO1 eluted at a position consistent with a dimer even in the absence of SDS. A minor peak of intact 9His-*rs*LAAO1 was detected at an elution volume corresponding to a molecular mass of 55 kDa. This peak has to be attributed to a monomer even though it is below the calculated molecular mass of 9His-*rs*LAAO1 (75 kDa). This difference could be due to a deviation from a spherical shape or to limited interaction with the matrix. The main peak was detected at an elution volume corresponding to 120 kDa, which is between twice the observed molecular mass of the monomer (110 kDa) and the calculated molecular mass of a dimer (150 kDa). The hydrodynamic radius calculated from PCS was larger than the theoretical radius, indicating that the protein had an elongated structure. Upon SDS activation, the apparent molecular mass is increased to 168 kDa. This size is still consistent with a dimer, which is most likely larger due to SDS conformational changes. SDS addition further increased the deviation from spherical shape according to PCS. A similar reduction in the elution volume in size-exclusion chromatography was observed for bean polyphenol oxidase upon SDS activation [32]. The authors conclude that the tetrameric configuration was not changed, but the increase in hydrodynamic radius was due to swelling or partial unfolding. Interestingly, SEC fractions of native 9His-*rs*LAAO1 with a slightly larger hydrodynamic radius than the dimer displayed a higher specific activity than the dimer when assayed without SDS activation. This difference was lost if the assay was performed after SDS activation. These data indicate that a small fraction of the 9His-*rs*LAAO1 dimer can spontaneously adopt a larger active conformation. 

SDS activation decreased the thermal stability of 9His-*rs*LAAO1. A similar reduction in thermal stability was observed for bean polyphenol oxidase [32] and latent mushroom tyrosinase [33] upon SDS activation. Treatment with low amounts of trypsin resulted in a stable 65 kDa fragment for native 9His-*rs*LAAO1, which was not observed for SDS-activated 9His-*rs*LAAO1. These data indicate that more trypsin sites were accessible due to SDS activation. Taken together, these data point towards a more open conformation of SDS-activated 9His-*rs*LAAO1. This conclusion is also supported by our spectroscopic experiments. SDS exposed some of the tryptophan residues of 9His-*rs*LAAO1 to a more hydrophilic environment according to changes in tryptophan fluorescence. The distance of FAD in 9His-*rs*LAAO1 to quenching amino acids increased due to SDS because the FAD fluorescence increased. In addition, lower concentrations of sulfites were sufficient to form adducts with FAD in SDS-activated 9His-*rs*LAAO1, pointing to an increase in accessibility to polar reagents and therefore a more hydrophilic environment of FAD. Both of these experiments point towards conformational changes in the active site due to SDS activation. Interestingly, changes in tryptophan and FAD fluorescence reached a plateau at 1 mM SDS upon increasing the concentration of SDS. Maximal activation of 9His-*rs*LAAO1 was also reached at 1 mM SDS [13]. Our conclusion is that the enzyme is quantitatively transformed into a defined active conformation at 1 mM SDS. However, the secondary structure was not significantly influenced according to CD spectroscopy in SDS-activated 9His-*rs*LAAO1. By contrast, SDS reduced the α-helical content in bean polyphenol oxidase [32]. Taken together, our data show that SDS caused changes in the tertiary structure of 9His-*rs*LAAO1 expressed in *E. coli* and increased the accessibility of the active site.

## 4. Materials and Methods 

### 4.1. Expression and Purification of 9His-rsLAAO1

MBP-P-9His-*rs*LAAO1 was expressed in the *E. coli* strain Arctic Express at 11 °C and purified via amylose resin followed by cleavage of the MBP tag with PreScission protease and further purification via a Ni^2+^-NTA column as described [13]. Protein concentrations were determined according to Bradford [34] using bovine serum albumin as standard. Samples were analyzed by SDS–PAGE and Coomassie staining for purity and size.

### 4.2. Enzyme Assay

Enzymatic activity was determined by measuring the initial rate of H_2_O_2_ production by a coupled peroxidase/*o*-dianisidine assay as described [13]. The assay mixture contained 10 mM l-arginine, 50 mM TEA/HCl buffer (pH 8.5), 0.2 mg∙mL^−1^ of *o-*dianisidine, 5 U mL^−1^ peroxidase and 9His-*rs*LAAO1 in limiting amounts. Reactions were carried out in 96-well plates at 30 °C in a Tecan Infinite 200 microplate reader at 436 nm. To determine temperature stability, 9His-*rs*LAAO1 was preincubated at the indicated temperatures and time periods. Native enzyme (0.1 mg∙mL^−1^) was supplemented with 1.5 mM SDS before the assay. After preincubation for 10 min, 2–5 µL of activated enzyme was used per assay (200 µL) resulting in a final concentration of SDS below 0.04 mM. This concentration of SDS did not influence the assay (Suppl. Figure S2).

### 4.3. Molecular Mass Determination by Size-Exclusion Chromatography

The native molecular mass of the purified 9His-*rs*LAAO1 was estimated by size-exclusion chromatography with an Ettan LC (GE Healthcare Life Sciences, Chicago, IL, USA) on calibrated columns of Superdex^®^ 200 Increase 10/300 GL (GE Healthcare Life Sciences, Chicago, IL, USA), using 50 mM NaH_2_PO_4_ buffer pH 8.2, 300 mM NaCl at a flow rate of 0.3 mL∙min^−1^. 1.5 mM SDS was added to the protein solution before chromatography to analyze the behavior of activated 9His-*rs*LAAO1. Dextranblue, thyroglobulin (669 kDa), ferritin (440 kDa), aldolase (158 kDa), bovine serum albumin (66 kDa), ovalbumin (44 kDa), cytochrome C (12.4 kDa) and aprotinin (6.5 kDa) were employed as standards to obtain calibration curves.

### 4.4. Photon Correlation Spectroscopy (PCS)

The angular-dependent PCS measurements were performed using a light scattering goniometer setup (LS Instruments, Fribourg, Switzerland) equipped with a HeNe laser at 632.8 nm (JDSU 1145P) and an attenuator to adjust the output power between 0 and 20 mW. The correlation functions were measured with a multiple τ digital correlator (Bridgewater, NJ, USA) and the angle-dependent mean relaxation rate $\bar{\Gamma }$ was observed in a scattering angle from 40° to 120°. The samples were measured in quartz cuvettes and slightly centrifuged before use to avoid contamination from dust or large aggregates. The temperature of the sample was regulated via a decalin-filled vat which was externally thermostatted with a refrigerated heating circulator (Julabo F25-ME, Julabo GmbH, Seelbach, Germany). The sample concentration was 2 mg∙mL^−1^ and the measurements were performed at 20 °C.

In photon correlation spectroscopy, the light scattering caused by particles in solution is measured and analysed. Temporal intensity fluctuations in the scattered light are evaluated using the formalism of correlation function [14]. From the measurement, the intensity autocorrelation function $g^{2}\left(\tau \right)$ is obtained and related to the electric field autocorrelation function $g^{1}\left(\tau \right)$ via the Siegert relation,

(1) $$g^{2}\left(\tau \right)=B\;\left[1+\beta \;{\left|g^{1}\left(\tau \right)\right|}^{2}\right],$$

where *B* is the baseline and β a coherence factor which depends on the scattering volume. For monodisperse samples of small molecules showing Brownian motion, the electric field autocorrelation function $g^{1}\left(\tau \right)$ is given by a single exponential function,

(2) $$g^{1}\left(\tau \right)=\exp\left(-\Gamma \;t\right),$$

with the relaxation rate $\Gamma =D^{T}q^{2}$, the translational diffusion coefficient $D^{T}$ and the magnitude of the scattering vector q,

(3) $$q=\frac{4\;\pi \;n}{\lambda }\cdot \sin\left(\theta /2\right),$$

with λ being the wavelength of the used radiation, n the refractive index and θ the scattering angle. A polydisperse sample can instead be described by a weighted sum of exponential decays.

(4) $$g^{1}\left(\tau \right)=\int_{0}^{\infty }G\left(\Gamma \right)\exp\left(-\Gamma \;t\right)d\Gamma$$

Here, G(Γ) represents the distribution of the relaxation rates. For an analysis of $g^{1}\left(\tau \right)$ the method of cumulants [35] or an inverse Laplace transformation using the FORTRAN program CONTIN [15,16] can be applied and provide the mean relaxation rate $\bar{\Gamma }$ of the relaxation rate distribution function. Since biological samples often show a non-monomodal distribution, the CONTIN analysis was the method of choice. 

For a dilute sample of non-interacting particles, the translational diffusion coefficient is related to the hydrodynamic radius via the Stokes–Einstein relation:

(5) $$D^{T}=\frac{k_{B}T}{6\pi \eta_{0}R_{h}},$$

where $k_{B}$ is the Boltzmann constant, $\eta_{0}$ the viscosity of the solvent and $R_{h}$ the hydrodynamic radius of a sphere which is hydrodynamically equivalent to the studied object in the solution.

### 4.5. Circular Dichroism Spectroscopy

Purified 9His-*rs*LAAO1 (0.25 mg∙mL^−1^) was dialyzed against 10 mM sodium phosphate buffer pH 8.0 containing 100 mM Na_2_SO_4_ and centrifuged for 5 min with 17,000× *g* to remove particles and aggregates. Spectra were recorded in a 1 mm quartz cuvette with a Jasco J-810 spectropolarimeter (JASCO, Easton, MD, USA) at 20 °C under continuous nitrogen flow. Three spectra were averaged. Mean molar ellipticity was determined as published [36]. The secondary structure was calculated with the CDSSTR algorithm used by the DichroWeb server [22].

### 4.6. Fluorescence Spectroscopy

Purified 9His-*rs*LAAO1 was dialyzed against 100 mM HEPES buffer pH 7.0 and centrifuged for 5 min with 17,000× *g* to remove particles and aggregates. Emission spectra were recorded in a quartz cuvette with a path length of 10 mm using a Perkin Elmer LB50B fluorescence spectrometer at (Perkin Elmer, Waltman, MA, USA) 22 °C. Excitation was at 295 nm for tryptophan fluorescence and at 450 nm for FAD fluorescence. The slit width for emission was set to 4.5 nm for tryptophan and 12.5 nm for FAD fluorescence. Spectra were not corrected for instrumental response.

### 4.7. Reactivity of FAD with Sulfite

Native 9His-*rs*LAAO1 (7.5 µM) or 9His-*rs*LAAO1 activated with 1.5 mM SDS (15 µM) were incubated with different amounts of sodium sulfite in 100 mM HEPES buffer pH 7.0 for 15 min at room temperature in the dark. Absorption spectra were recorded in a 10 mm cuvette with a Shimadzu UV-2450 UV/vis spectrophotometer (Shimadzu, Kyoto, Japan) at room temperature.

## Figures and Tables

**Table 1:** Summary of the theoretical hydrodynamic radii calculated from the molecular weight of 9His-*rs*LAAO1 (with and without SDS) and the obtained values of the anisotropy. SEC: size exclusion chromatography.

	Calculated	SEC (without SDS)	SEC with SDS
molecular weight	150 kDa (dimer)	120 kDa (dimer)	168 kDa (dimer)
theoretical radius	$R_{calc}^{theo}$ = 3.5 nm	$R_{SEC}^{theo}$ = 3.3 nm	$R_{SEC}^{theo}$ = 3.7 nm
theoretical hydrodynamic radius	$R_{h,calc}^{theo}$ = 3.8 nm	$R_{h,SEC}^{theo}$ = 3.6 nm	$R_{h,SEC}^{theo}$ = 4.0 nm
$\mathrm{experimental}\;R_{h,PCS}$	4.6 nm	4.6 nm	6.0 nm
$R_{h}/R_{h}^{theo}$	1.2	1.3	1.5

## Supplementary Materials

The following are available online.
