# Supplementary material for: Activation of Recombinantly Expressed l-Amino Acid Oxidase from Rhizoctonia solani by Sodium Dodecyl Sulfate
Source: Molecules. 2017 Dec 20;22(12):2272. doi: 10.3390/molecules22122272 (PMC6149798; doi:10.3390/molecules22122272)
Supplement: Supplementary file 1 [file molecules-22-02272-s001.pdf]

molecules

MDPI

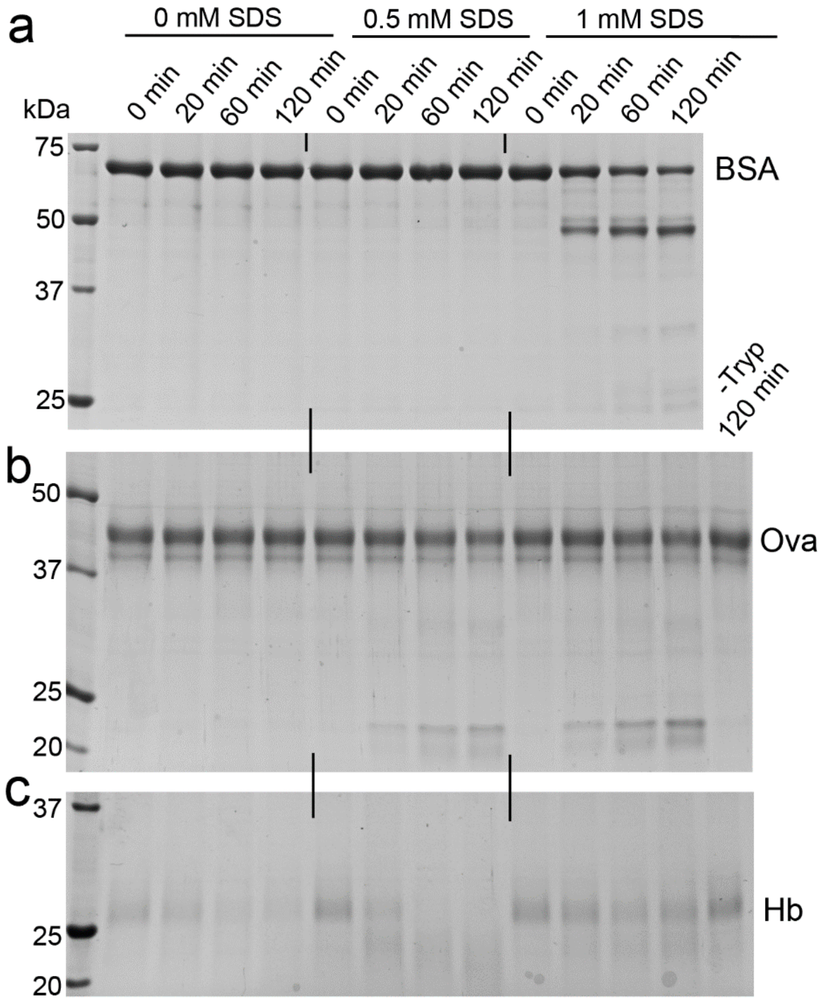

Supplementary Figure 1: Activity of trypsin in the presence of 0 mM, 0.5 mM or 1 mM SDS. (**a**) Bovine serum albumin (BSA), (**b**) ovalbumin (Ova) or (**c**) pig hemoglobin (Hb, all at 0.3 mg·mL^{-1}) were incubated with 0.3 µg·mL^{-1} trypsin in the same buffer as 9His-*rs*LAAO1 (100 mM HEPES pH 7.0, 50 mM NaCl) at 37 °C for the indicated periods of time in the presence of 0 mM, 0.5 mM or 1 mM SDS. A sample of ovalbumin and hemoglobin was also incubated without addition of trypsin (-Tryp) with 1 mM SDS for 120 minutes.

Bovine serum albumin was not proteolysed in 0 and 0.5 mM SDS. Some degradation was observed in 1 mM SDS indicating that the conformation of bovine serum albumin changed in a way that rendered it partially sensitive to trypsin. Ovalbumin was insensitive to trypsin in the absence of SDS. Smaller fragments in similar low amounts appeared in the presence of 0.5 mM and 1 mM SDS. Hemoglobin was sensitive to trypsin in the absence of SDS. Hemoglobin was fully degraded in the presence of 0.5 mM SDS. Only a partial loss of hemoglobin was observed in the presence of 1 mM SDS. Taken together these data indicate that trypsin was fully active in 0.5 mM SDS and partially active in 1 mM SDS.

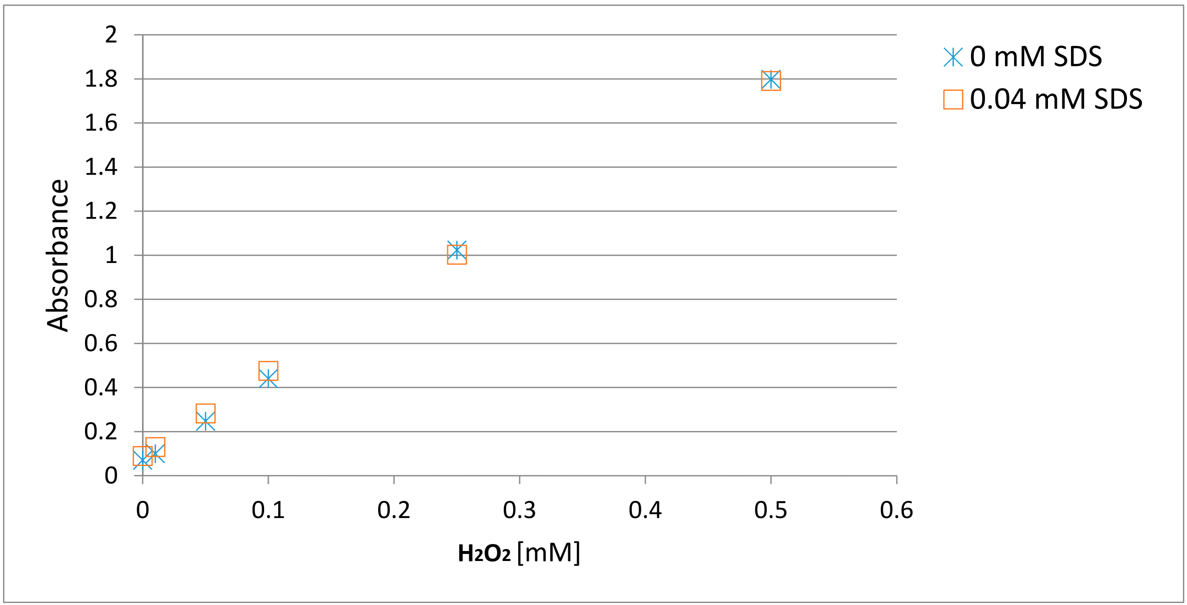

Supplementary Figure 2: Calibration curve with different concentrations of H_2O_2 revealed that 0.04 mM SDS did not influence the peroxidase/*o*-dianisidine assay.

Activation of 9His-*rs*LAAO1 with SDS resulted in a final concentration of SDS below 0.04 mM in the coupled peroxidase/*o*-dianisidine assay. Different concentrations of H_2O_2 were assayed with 0 mM or 0.04 mM SDS to study the effect of SDS on the assay system. The peroxidase (5 U mL^{-1}) was preincubated in the reaction buffer with 10 mM arginine in the absence or presence of 0.04 mM SDS for 10 minutes, *o*-dianisidine was added and the reaction started with 0.01–0.5 mM H_2O_2 in the absence of 9His-*rs*LAAO1. Absorbance was measured in a Tecan *infinite* 200 microplate reader at 436 nm after 5 minutes.
